# Efficacy and efficacy-influencing factors of stem cell transplantation on patients with Parkinson’s disease: a systematic review and meta-analysis

**DOI:** 10.3389/fneur.2024.1329343

**Published:** 2024-04-12

**Authors:** Jianli Zhao, Kang Qu, Shanshan Jia, Rong Yang, Ziting Cui, Jiajia Li, Peng Yu, Ming Dong

**Affiliations:** ^1^Department of Neurology and Neuroscience Center, The First Hospital of Jilin University, Changchun, China; ^2^Department of Ophthalmology, The Second Hospital of Jilin University, Changchun, China

**Keywords:** Parkinson, stem cell, transplantation, meta-analysis, clinical trial

## Abstract

**Background:**

Cell transplants as a treatment for Parkinson’s disease have been studied for decades, and stem cells may be the most promising cell sources for this treatment. We aimed to investigate whether stem cell transplantation contributes to the cure for Parkinson’s disease and the factors that may influence the efficacy for this therapy.

**Methods:**

PubMed, Embase, Cochrane Library, Web of Science, SinoMed, China National Knowledge Infrastructure (CNKI), China Science and Technology Journal Database (VIP), and ChinaInfo were thoroughly searched to find controlled trials or randomized controlled trials performing stem cell transplantation in patients with Parkinson’s disease. The pooled effects were analyzed to evaluate the weighted mean difference (WMD) with 95% confidence intervals.

**Results:**

Nine articles were identified including 129 individuals. Stem cell transplantation was an effective treatment for Parkinson’s disease (WMD = −14.86; 95% CI: −16.62 to −13.10; *p* < 0.00001), with neural stem cells, umbilical cord mesenchymal stem cells (UCMSCs), and bone marrow mesenchymal stem cells (BMMSCs) being effective cell sources for transplantation. Stem cell transplantation can be effective for at least 12 months, but its long-term effectiveness remains unknown due to the limited studies monitoring patients for more than 1 year, not to mention decades.

**Conclusion:**

Data from controlled trials suggest that stem cell transplantation as a therapy for Parkinson’s disease can be effective for at least 12 months. The factors that may influence its curative effect are time after transplantation and stem cell types.

**Systematic review registration:**

(Registration ID: CRD42022353145).

## Introduction

Parkinson’s disease is a progressive neurodegenerative disease characterized by psychiatric disturbances (depression and anxiety), cognitive problems (cognitive decline), movement difficulties (tremor, stiffness, and slowness), and motor complications (dyskinesia) associated with medication use; the Unified Parkinson’s Disease Rating Scale (UPDRS) was developed to estimate the severity of this disease. Treatment for Parkinson’s disease involves pharmacologic approaches, typically with levodopa preparations prescribed with or without other medications, and nonpharmacologic approaches, such as exercise and physical, occupational, and speech therapies (1); however, neuropathological evidence suggests that Parkinson’s disease is characterized by a selective loss of dopaminergic neurons in the substantia nigra pars compacta, with widespread involvement of other central nervous system structures and peripheral tissues (2). Farzane Sivandzade pointed out that the currently available treatment options are insufficient in arresting the neurodegenerative processes; hence, stem cell transplantation is preferred to enable neuro-restoration in patients with this condition (3).

Cell transplants as a treatment for Parkinson’s disease have been studied for decades; stem cells are currently used as cell sources in the treatment of this condition (4). Initially, people use tissue or dopaminergic neuron precursor cells isolated from a fetus (5–7); however, the results could be variable when people use fetal tissue-derived cells or tissues for transplant. Fetal tissues or cells have great heterogeneity and have difficulties in quality control; this maybe the reason why negative results were obtained in double-blind, sham-controlled clinical trials conducted by Freed and Olanow (7, 8). Therefore, the development of stem cell biology led to the discovery of other cell sources for transplantation, which helped to partially address the problems associated with the availability of fetal tissue-derived cells and the impossibility of standardization, resulting in a more steady course for this therapy (4). Potential cell sources include homogenous stem cells derived from the human body (BMMSCs and UCMSCs), dopamine cells derived from embryonic stem cells, and dopamine cells derived from induced pluripotent stem cells (4, 9, 10). However, because early tissue transplantation had such a varied outcome, most researchers in recent years have increasingly preferred to begin with transplanting homogeneous cells, and the majority of studies utilizing populations comprising stem cells are in the preclinical stage. Others could be worried about the immunological regulatory cells that populations including stem cells, such blood cells from different donors, may introduce and the possibility of graft-versus-host disease. Hence, the efficacy of homogeneous stem cells for patients with Parkinson disease is the main focus of our meta-analysis.

Because the loss of dopaminergic neurons is considered as the main cause for the key motor symptoms and signs of Parkinson’s disease, many researchers think replacing them with stem cell-derived dopamine cells can relieve patients with Parkinson (4). However, Parkinson disease has not only key motor symptoms, but also a multi-system disorder with extranigral pathology or symptoms that are unresponsive to levodopa, and evidences from genetic, pharmacological, immunological, neuroimaging, epidemiological studies support neuroinflammation important in progress of Parkinson disease (11). Therefore, our meta-analysis focuses on the results of studies using less differentiated stem cells as transplanting cell sources to see if modulation of the inflammatory and immune environment from stem cells transplanted can be effective.

The different stem cells used for clinical transplantation include neural stem cells (12–14), BMMSCs (15, 16), UCMSCs (17–20), adipose derived neural progenitor cells (12), and human retinal pigment epithelium cells (21); meanwhile, the different methods of transplantation can be categorized as follows: intraventricular injection, intravascular injection, and intrathecal injection. Various trials can conduct transplantation differently using the same method; in the clinical trials conducted by Purwati, an Ommaya reservoir was inserted into the ventricle, and booster implantation of stem cells were performed 1 to 2 months after (22). Others may only perform injections into the ventricle once. Significant differences were also observed in terms of the area where the stems cells were injected; that is, some performed intravenous injection of stem cells, while others performed arterial injection targeting the anterior or posterior brain circulation.

Considering the different methods of administering the treatment, we examined the influence of these differences and determined whether stem cell transplantation can benefit patients with Parkinson’s disease. Therefore, we conducted a meta-analysis to evaluate the efficacy of stem cell transplantation for Parkinson’s disease and the influencing factors.

## Materials and methods

### Methods and search strategy

The present meta-analysis was conducted in accordance with the criteria reported by the Preferred Reporting Items for Meta-Analyses group. This review was conducted in accordance with the 2020 Preferred Reporting Items for Systematic Reviews (23) and was registered in the International Prospective Register of Systematic Reviews (registration ID: CRD42022353145). The study did not require ethics committee approval owing to its non-experimental design and search strategy. English databases (PubMed, Embase, Web of Science, and Cochrane Library) and Chinese databases (CNKI, China Info, VIP, and Sino Med) were searched by two reviewers (ZC and JL) to find eligible studies published from the inception of databases to August 25, 2022. The references of selected articles were screened independently by two workers (RY and SJ) to identify additional studies; then, the final list of literature that should be included in the meta-analysis was discussed to resolve all disagreements. The terms used in searching PubMed are summarized in Table 1.

**Table 1:** Terms used in searching PubMed.

Search number	Query
#1	“Parkinson Disease”[Mesh]
#2	(((((((Idiopathic Parkinson’s Disease[Title/Abstract]) OR (Lewy Body Parkinson’s Disease[Title/Abstract])) OR (Parkinson’s Disease[Title/Abstract])) OR (Parkinson Disease[Title/Abstract])) OR (Idiopathic Parkinson Disease[Title/Abstract])) OR (Lewy Body Parkinson Disease[Title/Abstract])) OR (Primary Parkinsonism[Title/Abstract])) OR (Paralysis Agitans[Title/Abstract])
#3	#1 OR #2
#4	“Stem Cells”[Mesh]
#5	((((stem cell*[Title/Abstract]) OR (Progenitor Cell*[Title/Abstract])) OR (Mother Cell[Title/Abstract])) OR (Colony-Forming Unit*[Title/Abstract])) OR (Colony Forming Unit*[Title/Abstract])
#6	#4 OR #5
#7	((((((randomized controlled trial[Publication Type]) OR (controlled clinical trial[Publication Type])) OR (randomized[Title/Abstract])) OR (controlled[Title/Abstract])) OR (trial[Title/Abstract])) OR (random[Title/Abstract])) OR (placebo[Title/Abstract])
#8	#3 AND #6 AND #7

### Assessment of eligibility

Studies that fulfilled the following criteria were considered eligible for the meta-analysis: (1) studies that included patients diagnosed with Parkinson’s disease and whose diagnosis were clearly and correctly described, (2) controlled trials, (3) studies that performed a stem cell transplantation as an intervention using stem cells collected from a human donor, and (4) studies whose primary outcomes were UPDRS, mean difference (MD), and standard deviation of UPDRS calculated from the data or figures provided. We excluded studies that included patients with Parkinsonism or Parkinson’s-plus syndromes. Studies that only provided the scores in Part III of the UPDRS or the percentage of improvement in UPDRS, did not report the total score, and did not use homogenous stem cells as cell sources for transplantation were also excluded. Duplicate studies, conference, reviews, case reports, and meta-analysis were removed. Three reviewers (JLZ, SSJ and YR) independently performed the study selection.

### Data extraction

Data extraction was performed independently by two investigators. The following data were extracted from the included studies: name of first author, publication year, follow-up year, population age range, sample size, cell types for transplantation, route of transplantation, and UPDRS as main outcome. Discrepancies were resolved by discussing the issues between the two investigators or by consulting a third investigator.

### Quality assessment

Quality assessment for randomized controlled trials was performed using the Jadad scale (24), which is a scale used for the assessment of randomized controlled studies in meta-analyses; scores ranged from 0 to 7, with higher scores indicating a higher quality. Studies that obtained a score of 4 or higher were defined as high-quality studies. Quality assessment of controlled trials was performed according to the methodological index for non-randomized studies, the final version of which contained 12 items (including study aim, data collection, follow-up time, and baseline equivalence of groups). It is used for the assessment of controlled studies in meta-analyses; scores ranged from 0 to 24, with higher scores indicating a higher quality. Hence, studies that obtained a score of 13 or higher were defined as high-quality studies (25). Two reviewers independently evaluated the quality of all included studies.

### Statistical analyses

Review Manager 5.4 (Revman 5.4) and Stata software version 16.0 (StataCorp, College Station, TX, United States) were used for performing all statistical analyses. WMD and 95% CI were used as effect sizes, and a forest plot was made using Revman 5.4. Cochran’s Q test and the *I*^2^ statistic were used to evaluate the heterogeneity across studies. A *p* value of <0.1 or an *I*^2^ value of >50% indicated a significant heterogeneity. The random effects model was employed. A subgroup analysis was conducted according to the cell types, route of transplantation, and tracking time after transplantation to explore the potential source of heterogeneity. Sensitivity analysis and publication bias were assessed using Stata 16.0 (StataCorp, College Station, TX, United States); sensitivity analysis was performed to evaluate the stability of the results by removing one study at a time. Meanwhile, publication bias was evaluated using Egger’s rank test and was presented using a funnel plot.

## Results

### Search results and study characteristics

**Figure 1:**
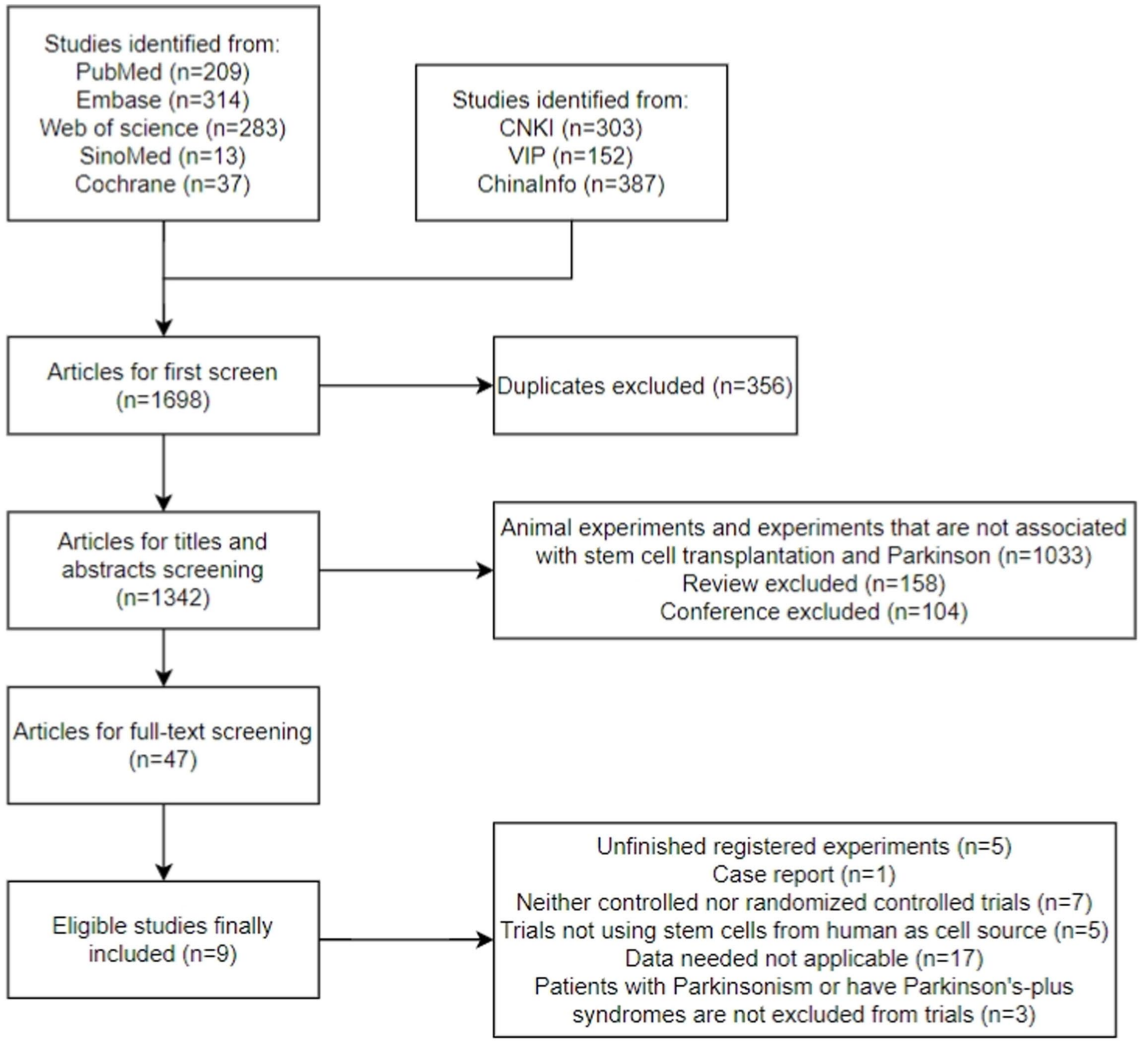
Flow diagram of the study selection process.

A total of 1,698 articles were retrieved from all databases: 302 from CNKI, 152 from VIP, 387 from ChinaInfo, 209 from PubMed, 37 from Cochrane, 13 from SinoMed, 314 from Embase, and 283 from Web of Science (Figure 1). We excluded 356 duplicates using endnotes and then screened the titles and abstracts of the remaining articles; we excluded 158 reviews and meta-analysis, 104 conferences, and 1,033 articles that were irrelevant. A total of 47 articles were subjected to a full-text review, and 38 articles that were not completely registered, with irrelevant research topic, or whose data were not applicable in the current meta-analysis were excluded.

Finally, a total of nine articles (13–15, 17–21, 26) were included, all of which were controlled trials. All studies obtained a quality score of more than 12 according to MINORS. The baseline characteristics and quality assessment results of all nine articles are summarized in Table 2.

**Table 2:** Characteristics of the studies included in this meta-analysis.

First Author/Year	country	Follow-up time	Age, mean ± SD/ Age range (years)	Women n (%)	Study design	Sample size	Experiment group		Control group	Assessment point of UPDRS	Minors
							Stem cell type	Transplantation route			
Xiaoqun et al. (2013) (13)	China	60 days	62.0 ± 4.2	5 (45.5%)	clinical trial	22	Fetal tissue-derived neural stem cell	Intrathecal injection	Levodopa	Baseline, 1, 3, 7, 30 and 60 days	14
Lige and Zengmin (2014) (14)	China	2 years	57.3 ± 9.1	6 (28.6%)	clinical trial	21	Fetal tissue-derived neural stem cell	Intraventricular injection	Levodopa	Baseline, 2 years	15
Schiess et al. (2021) (15)	USA	52 weeks	66.4 ± 5.9	3 (60%)	phase I study	5	Bone marrow mesenchymal stem cell	Intravascular injection	Dopaminergic regimen	Baseline, 3, 12, 24, and 52 weeks	17
Sun et al. (2016) (17)	China	3 weeks	65.3 ± 2.7	4 (40%)	clinical trial	10	Umbilical cord mesenchymal stem cell	Intravascular injection	Levodopa	Baseline, 1 month	16
Yan et al. (2014) (18)	China	1 month	63.4 ± 7.6	7 (46.7%)	clinical trial	15	Umbilical cord mesenchymal stem cell	Intravascular injection	Drug treatment	Baseline, 1 month	14
Yun et al. (2011) (19)	China	1 month	58.4 ± 8.7	4 (50%)	clinical trial	8	Umbilical cord mesenchymal stem cell	Intravascular injection	Levodopa	Baseline, 1 month	14
Aili (2013) (20)	China	6 months	67.1 ± 5.3	4 (44.4%)	Clinical trial	9	Umbilical cord mesenchymal stem cell	Intrathecal injection	Levodopa	Baseline, 28 days, 6 months	16
Yin et al. (2012) (21)	China	36 months	66.0 ± 11.4	4 (44.4%)	pilot clinical trial	9	Human retinal pigment epithelium cell	Intraventricular injection	Levodopa	Baseline, 3, 6, 12, 24 and 36 months	13
Dapeng et al. (2013) (26)	China	12 weeks	45–66	14 (46.7%)	clinical trial	30	Umbilical cord mesenchymal stem cell	Intrathecal injection	Compound levodopa	Baseline, 4 and 12 weeks	15

### Effectiveness of stem cell transplantation in Parkinson’s disease

**Figure 2:**
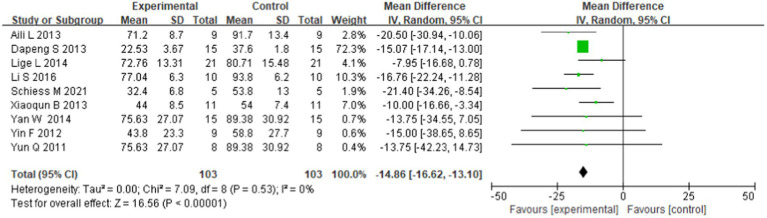
Forest plot of UPDRS after the longest follow-up time of stem cell transplantation for Parkinson’s disease reported in all nine studies selected.

Nine eligible studies reported changes in UPDRS after stem cell transplantation in patients with Parkinson’s disease (WMD = −14.86; 95% CI: −16.62 to −13.10; *p* = 0.53; *I*^2^ = 0%), showing that stem cell transplantation is effective for Parkinson’s disease (Figure 2) in the fixed effects model.

### Subgroup analysis based on cell type

**Figure 3:**
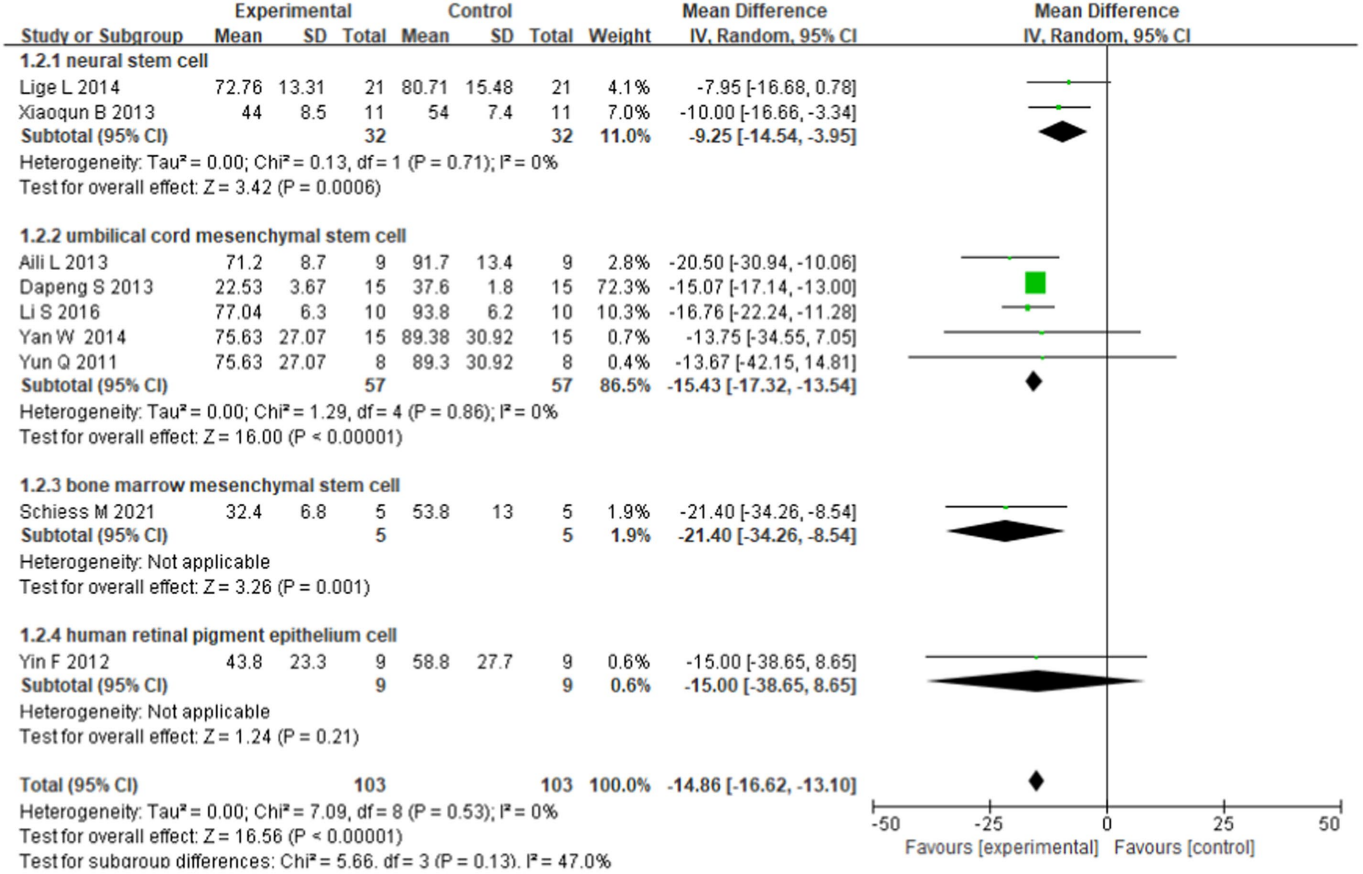
Forest plot of the subgroup analysis based on cell type.

For each subgroup, the fixed effects model with low heterogeneity was used to conduct this subgroup analysis. Results showed that both neural stem cells (WMD = −9.25; 95% CI: −14.54 to −3.95) and UCMSCs (WMD = −15.43; 95% CI: − 17.32 to −13.54) were effective treatments for Parkinson’s disease. Only one study used BMMSCs for transplantation and was also effective in treating Parkinson’s disease (MD = −21.40; 95% CI: − 34.26 to −8.54). Another study used human retinal pigment epithelium cells for transplantation and seemed ineffective (MD = −15.00; 95% CI: − 38.65 to 8.65). Differences were observed in the combined effects of the subgroups, but the differences were not significant (chi-square = 5.66, degree-of-freedom (df) = 3 (*p* = 0.13); *I*^2^ = 47.0%; Figure 3).

### Subgroup analysis based on transplantation route

**Figure 4:**
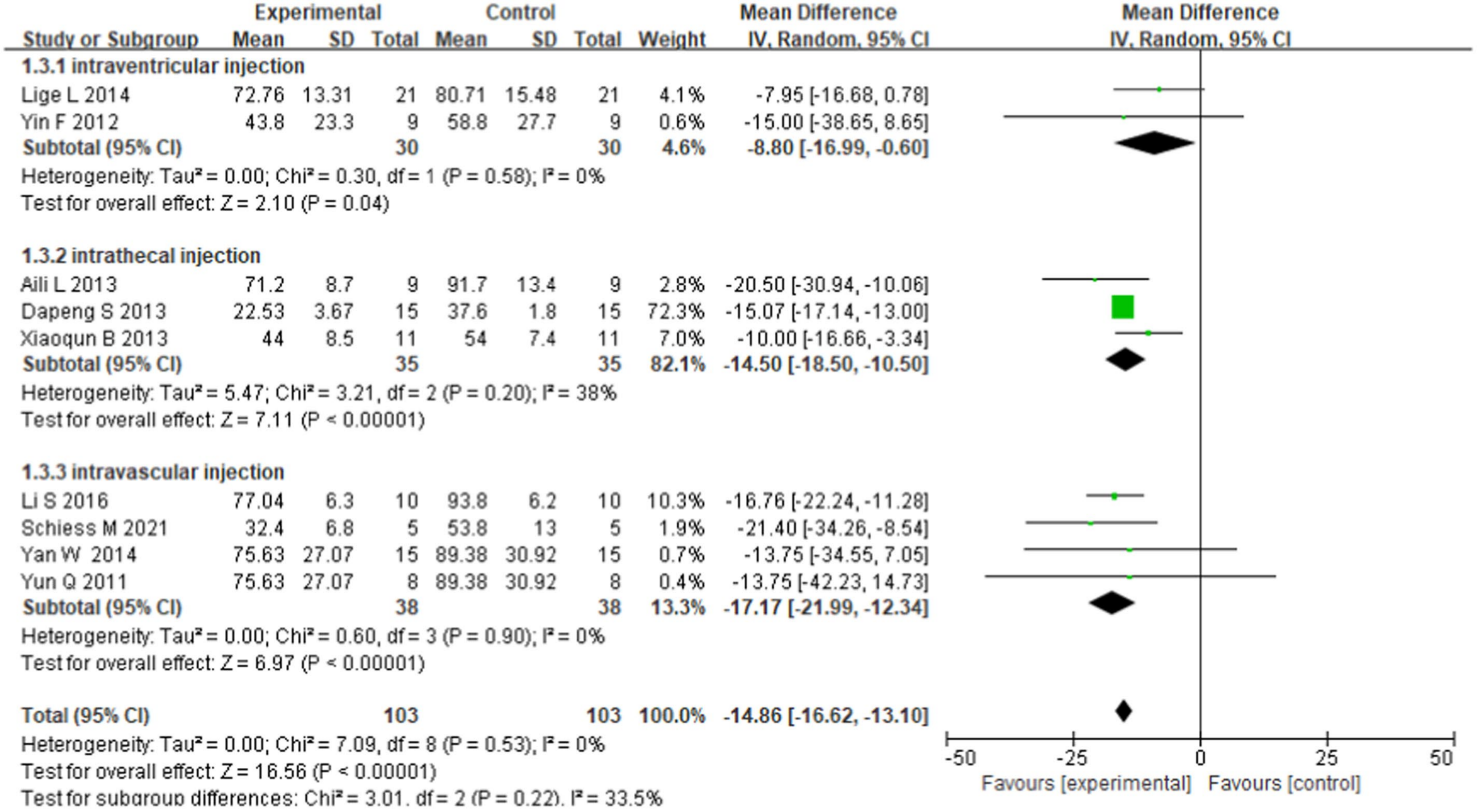
Forest plot of subgroup analysis based on transplantation route.

The degree of heterogeneity decreased in the intraventricular injection subgroup and intravascular injection group, but was higher (*I*^2^ = 38%) in the intrathecal injection subgroup. We used the fixed effects model in this subgroup analysis. The results were as follows: intraventricular injections were effective (WMD = −8.80; 95% CI: −16.99 to −0.60; *p* = 0.58; *I*^2^ = 0%), intrathecal injections were effective (WMD = −14.50; 95% CI: −18.50 to −10.50; *p* = 0.20; *I*^2^ = 38%), and intravascular injections were effective (WMD = −17.17; 95% CI: −21.99 to −12.34; *p* = 0.90; *I*^2^ = 0%). Differences were observed in the combined effects of the subgroups, but the differences were not significant (chi-square = 3.01, df = 2 (*p* = 0.22); *I*^2^ = 33.5%; Figure 4).

### Subgroup analysis based on tracking time

**Figure 5:**
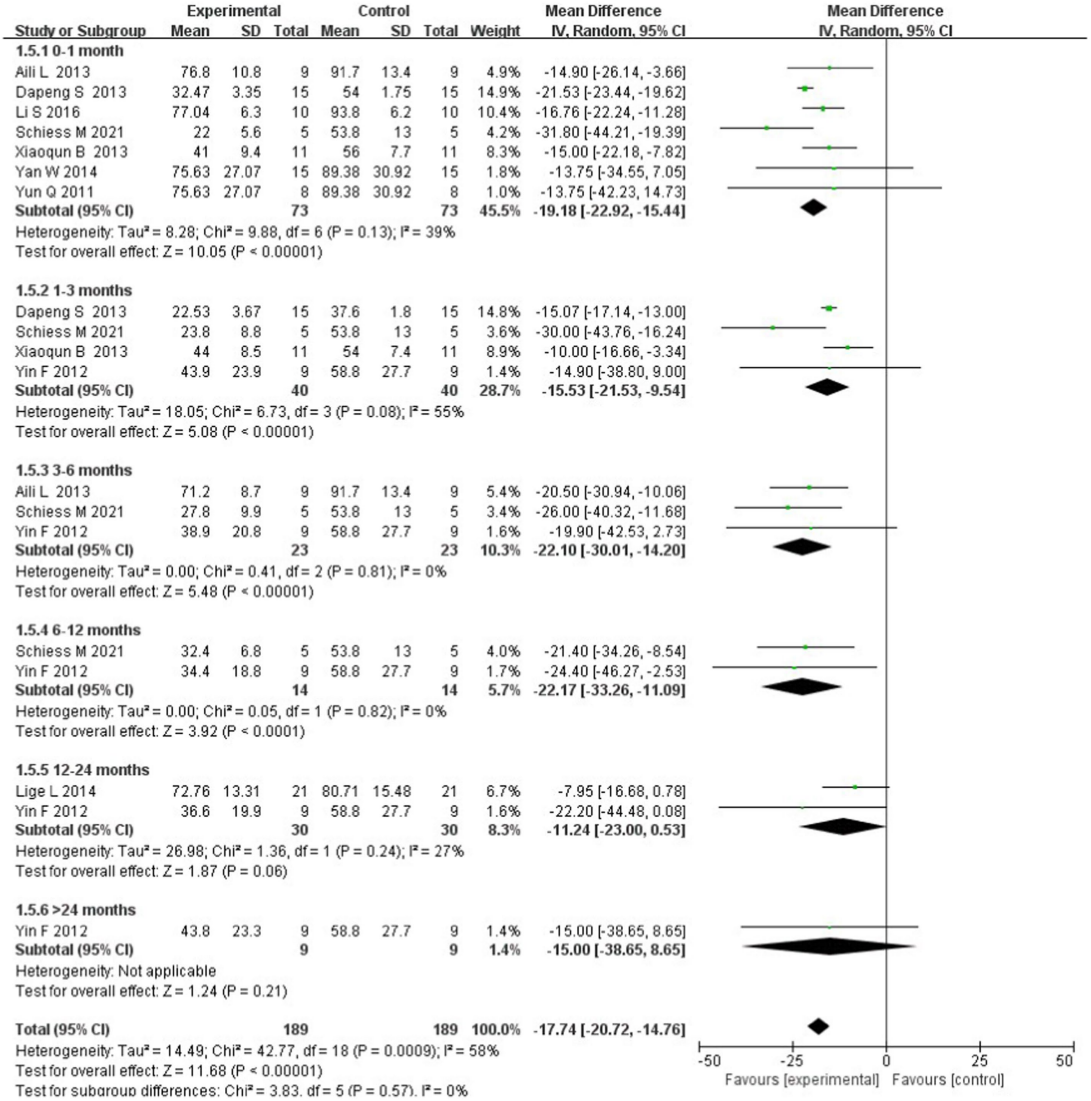
Forest plot of the subgroup analysis based on tracking time after stem cell transplantation.

A subgroup analysis was performed to explore the changes in the treatment effectiveness over time. Most studies demonstrated two to three endpoints at various time points: 0–1 month after intervention was effective (WMD = −19.18; 95% CI: −22.92 to −15.44; *p* = 0.13; *I*^2^ = 39%), 1–3 months after intervention was effective (WMD = −15.53; 95% CI: −21.53 to −9.54; *p* = 0.08; *I*^2^ = 55%), 3–6 months after intervention was effective (WMD = −22.10; 95% CI: −30.01 to −14.20; *p* = 0.81; *I*^2^ = 0%), 6–12 months after intervention was effective (WMD = −22.17; 95% CI: −33.26 to −11.09; *p* = 0.82; *I*^2^ = 0%), and 12–24 months after intervention was effective (WMD = −11.24; 95% CI: −23.00 to 0.53; *p* = 0.24; *I*^2^ = 27%); only one trial showed results after 24 months and reported that stem cell transplantation was ineffective (WMD = −15.00; 95% CI: −38.65 to 8.65). Significant differences were found between the subgroups [chi-square = 3.83, df = 5 (*p* = 0.57); *I*^2^ = 0%]; therefore, stem cell transplantation is effective for at least 12 months after the intervention (Figure 5).

### Sensitivity analysis

**Figure 6:**
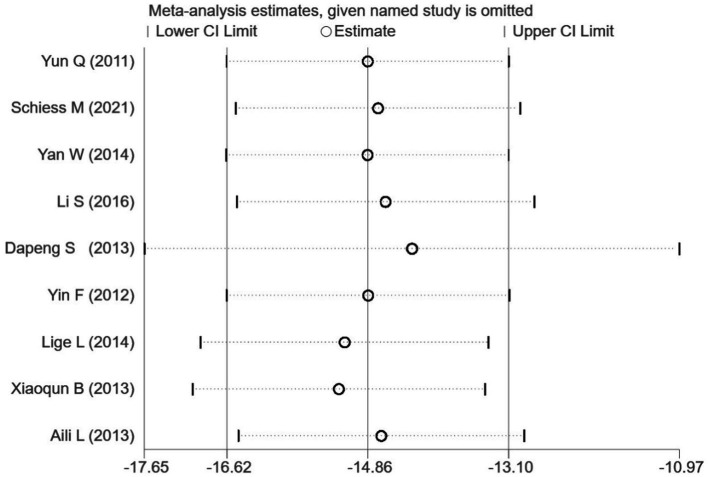
Forest plot of the sensitivity analysis by excluding one study each time and the pooling estimate for the remaining studies.

Stata 16.0 was used to perform a sensitivity analysis by excluding one study each time. None of the studies had an efficient impact on the pooled effect size (Figure 6); this finding indicates that the results of the present meta-analysis are reliable and stable.

### Publication bias

**Figure 7:**
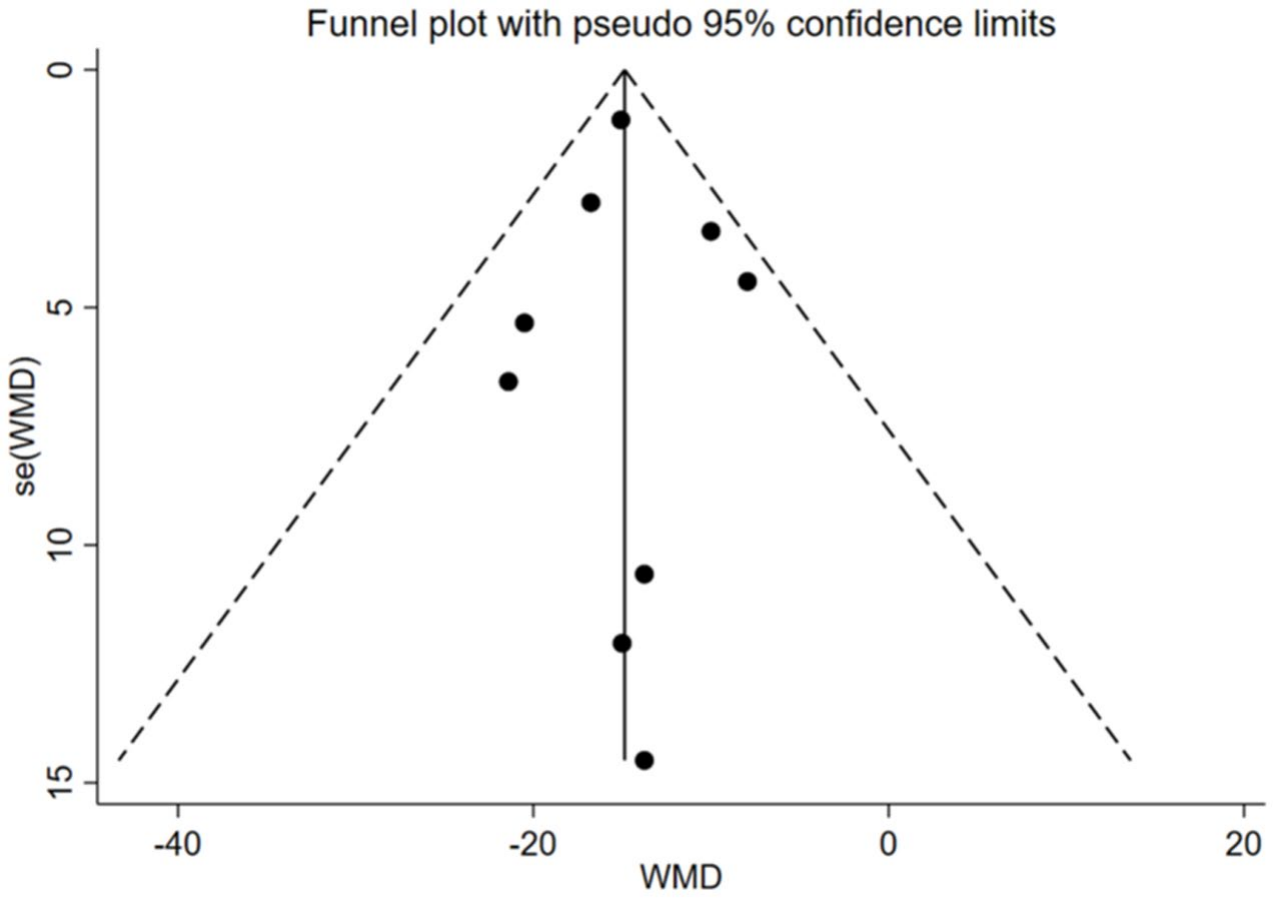
Funnel plot of the WMD vs. standard error of WMD for all nine included studies.

A funnel plot was created to show the publication bias (Figure 7), and Egger’s test was performed using Stata 16.0. Results showed no obvious publication bias in our meta-analysis based on the symmetrical funnel plot drawn and results of Egger’s (*p* = 0.849) test.

## Discussion

Our meta-analysis is the first to integrate the data from pilot clinical trials on stem cell transplantation as treatment for Parkinson’s disease and is considered meaningful for further experiments conducted in this field. Results showed that stem-cell transplantation is an effective therapy for Parkinson’s disease (WMD = −14.54; 95% CI: −16.32 to −12.77; *p* < 0.00001) and is influenced by cell type used for transplantation, and duration of effectiveness after transplantation.

In cell type subgroups, BMMSCs obtained the highest WMD value, and UCMSCs ranked second; meanwhile, the retinal pigment epithelium cells may be ineffective when used as treatment for Parkinson’s disease. Although BMMSCs obtained the highest MD value, the sample size was relatively small to conclude that these cells were the most effective treatments; the findings were obtained in a previous study using retinal pigment epithelium cells for transplantation. Hence, more studies are warranted to confirm whether retinal pigment epithelium cells are indeed ineffective. BMMSCs and UCMSCs are both MSCs. MSCs from various sources have low immunogenicity and immunomodulatory abilities enabling them to be transplanted in an autologous or allogeneic manner. In addition, they are antiapoptotic, multidirectional, and easy to collect (27). Generally, MSCs may be preferred for stem cell transplantation in patients with Parkinson’s disease; however, the most effective subtype of MSCs for Parkinson’s disease can only be confirmed once further clinical trials are completed.

In the subgroup analysis, intravascular injections may show higher effectiveness compared with intraventricular injections. One possible reason for this is that the small sample of patients included. Another possible reason for this is that more stem cells can be injected intravascularly than intraventricularly. In order to achieve good clinical effects, sufficient amounts of tissues are required (4), and this may also be true for stem cells. Several studies examining the brains of patients with Parkinson’s disease receiving fetal neural grafts over a decade prior to death have shown that some of the grafted dopamine neurons exhibited the production of Lewy bodies and increased the levels of soluble α-synuclein; more detailed follow-up studies reported that some of the grafted neurons progressively expressed reduced levels of dopamine transporters and tyrosine hydroxylase; this result further supports the notion that the disease process directly impacts the grafted cells (28–32). Therefore, improving the microenvironment inside the patients’ brain may also be important; this means that the modulation in inflammatory and immune environment done by pluripotent stem cells can be useful for Parkinson patients even after their dopaminergic cell transplantation.

One variable affecting efficacy is the duration following transplantation. Result showed that the effectiveness of stem cells after transplantation lasts for 12 months, but transplantation can become ineffective after 12 months. A different work also yields a similar conclusion. Cell therapy, according to Wang and colleagues, at least decreased UPDRS scores in the 12-month follow-up group (33). Similar findings imply that most transplanted cells have a maximum survival period of 12 months. Nevertheless, none of the included studies had followed up on the stem cells’ efficacy long enough to assess the long-term results.

Although our meta-analysis focuses on effectiveness of pluripotent stem cells for Parkinson disease, we still looking forward to the upcoming outcomes of studies undergoing with dopaminergic cell, for example, TRANSEURO study or studies conducted by Takahashi’s group (34, 35). The different outcomes between dopaminergic cell transplantation and pluripotent stem cell transplantation can help us figure out a better way for treatment. Besides, we are eager to investigate the influence of cell dose if studies had reported it in the same unit. We need further trials to come to a more scientific conclusion, even if the study by Schiess M (15) indicated that the group receiving the highest dose had the greatest drop in the overall UPDRS. More data will be available for assessment soon, and we are interested in exploring how cell dose affects stem cell transplantation’s efficacy.

Our meta-analysis has several limitations. Most of our studies were controlled trials without randomized method and blinded method, which was common in invasive treatment experiments and may contribute to the occurrence of bias. Besides, studies only reporting the scores of UPDRS-III were not included; however, stem cell transplantation is believed to improve the motor symptoms of Parkinson’s disease. Hence, the endpoints of the experiments should be comprehensively evaluated. In addition, the sample size of our meta-analysis was relatively small as all analyzed trials only included a few participants; however, the heterogeneity of our meta-analysis was low. Moreover, the follow-up time was not sufficiently long; considering that the stem cell transplantation is a newly developed method, studies with long-term follow-up are needed.

Generally, stem cell transplantation can be effective for Parkinson’s disease for at least 12 months, and MSCs are recommended as cell sources; however, this finding cannot be confirmed owing to the lack of systematic long-term follow-up of the outcomes of sham surgery-controlled trials.

## Data availability statement

The original contributions presented in the study are included in the article/Supplementary material, further inquiries can be directed to the corresponding authors.

## Author contributions

JZ: Conceptualization, Formal analysis, Writing – original draft. KQ: Methodology, Writing – review & editing. SJ: Formal analysis, Writing – review & editing. RY: Data curation, Writing – review & editing. ZC: Data curation, Writing – review & editing. JL: Data curation, Writing – review & editing. PY: Supervision, Writing – review & editing. MD: Project administration, Supervision, Writing – review & editing.
