# Supplementary material for: Efficacy and efficacy-influencing factors of stem cell transplantation on patients with Parkinson’s disease: a systematic review and meta-analysis
Source: Front Neurol. 2024 Apr 12;15:1329343. doi: 10.3389/fneur.2024.1329343 (PMC11045895; doi:10.3389/fneur.2024.1329343)
Supplement: Supplementary file 1 [file Table_1.docx]

| **Section and Topic** | **Item #** | **Checklist item** | **Location where item is reported** |
| --- | --- | --- | --- |
| **TITLE** | | |  |
| Title | 1 | The study was defined as systematic review and meta-analysis. | Page 1 |
| **ABSTRACT** | | |  |
| Abstract | 2 | The structed abstract includes Background and purpose, Methods, Results and Conclusion. | Page 1 |
| **INTRODUCTION** | | |  |
| Rationale | 3 | Described in the Introduction. | Page 2 |
| Objectives | 4 | Stated in the Introduction. | Page 2 |
| **METHODS** | | |  |
| Eligibility criteria | 5 | Described in the Assessment of eligibility of Materials and methods. | Page 2-3 |
| Information sources | 6 | Described in the Methods and research strategy of Materials and methods. | Page 2 |
| Search strategy | 7 | Described in the Methods and research strategy of Materials and methods. | Page 3 |
| Selection process | 8 | Described in the Data extraction of Materials and methods. | Page 2-3 |
| Data collection process | 9 | Described in the Data extraction of Materials and methods. | Page 3 |
| Data items | 10a | Described in the Data extraction of Materials and methods. | Page 3 |
|  | 10b | Described in the Data extraction of Materials and methods. | Page 3 |
| Study risk of bias assessment | 11 | Described in the Quality assessment of Materials and methods. | Page 3 |
| Effect measures | 12 | Described in the Statistical analysis of Materials and methods. | Page 3 |
| Synthesis methods | 13a | Describe in the Assessment of eligibility. | Page 2-3 |
|  | 13b | Described in the Statistical analysis of Materials and methods. | Page 3 |
|  | 13c | Described in the Statistical analysis of Materials and methods. | Page 3 |
|  | 13d | Described in the Statistical analysis of Materials and methods. | Page 3 |
|  | 13e | Described in the Statistical analysis of Materials and methods. | Page 3 |
|  | 13f | Described in the Statistical analysis of Materials and methods. | Page 3 |
| Reporting bias assessment | 14 | Described in the Statistical analysis of Materials and methods. | Page 3 |
| Certainty assessment | 15 | Described in the Statistical analysis of Materials and methods. | Page 3 |
| **RESULTS** | | |  |
| Study selection | 16a | Described in the Search results and study characteristics. | Page 3-4 |
|  | 16b | Described in the Search results and study characteristics. | Page 3-4 |
| Study characteristics | 17 | Described in the Search results and study characteristics. | Page 5 |
| Risk of bias in studies | 18 | Described in the Search results and study characteristics. | Page 5 |
| Results of individual studies | 19 | Described in Effectiveness of stem cell transplantation for Parkinson, Subgroup analysis based on cell type, Subgroup analysis based on transplantation route, Subgroup analysis based on tracking time. | Page 3,4,6 |
| 5-6Results of syntheses | 20a | Described in Effectiveness of stem cell transplantation for Parkinson, Subgroup analysis based on cell type, Subgroup analysis based on transplantation route, Subgroup analysis based on tracking time. | Page 5,6 |
|  | 20b | Described in Effectiveness of stem cell transplantation for Parkinson, Subgroup analysis based on cell type, Subgroup analysis based on transplantation route, Subgroup analysis based on tracking time. | Page 3,4,6 |
|  | 20c | Described in Effectiveness of stem cell transplantation for Parkinson, Subgroup analysis based on cell type, Subgroup analysis based on transplantation route, Subgroup analysis based on tracking time. | Page 3,4,5,6 |
|  | 20d | Described in the Sensitivity analysis. | Page 6 |
| Reporting biases | 21 | Described in the Publication bias | Page 6 |
| Certainty of evidence | 22 | Described in Effectiveness of stem cell transplantation for Parkinson, Subgroup analysis based on cell type, Subgroup analysis based on transplantation route, Subgroup analysis based on tracking time. | Page 3,4,6 |
| **DISCUSSION** | | |  |
| Discussion | 23a | Described in the Discussion. | Page 6,7 |
|  | 23b | Described in the Discussion. | Page 7,8 |
|  | 23c | Described in the Discussion. | Page 7,8 |
|  | 23d | Described in the Discussion. | Page 6,7,8 |
| **OTHER INFORMATION** | | |  |
| Registration and protocol | 24a | Described in the Methods and search strategy. | Page 2 |
|  | 24b | The protocol is described in the Methods and search strategy. | Page2-3 |
|  | 24c | No amendment explained. | - |
| Support | 25 | Described in the Acknowledgments. | Page 9 |
| Competing interests | 26 | Described in the Conflict of interest. | Page 9 |
| Availability of data, code and other materials | 27 | Described in the Data availability statement. | Page 8 |

*From:*  Page MJ, McKenzie JE, Bossuyt PM, Boutron I, Hoffmann TC, Mulrow CD, et al. The PRISMA 2020 statement: an updated guideline for reporting systematic reviews. BMJ 2021;372:n71. doi: 10.1136/bmj.n71

For more information, visit: <http://www.prisma-statement.org/>
